# Twelve quick tips for designing sound dynamical models for bioprocesses

**DOI:** 10.1371/journal.pcbi.1007222

**Published:** 2019-08-22

**Authors:** Francis Mairet, Olivier Bernard

**Affiliations:** 1 Ifremer, Physiology and Biotechnology of Algae laboratory, Nantes, France; 2 Côte d’Azur University, INRIA, BIOCORE, Sophia-Antipolis Cedex, France; 3 Sorbonne University, CNRS, LOV, Villefranche-sur-mer, France; 4 ENERSENSE, Department of Energy and Process Engineering, NTNU, Trondheim, Norway; University of Toronto, CANADA

## Abstract

Because of the inherent complexity of bioprocesses, mathematical models are more and more used for process design, control, optimization, etc. These models are generally based on a set of biochemical reactions. Model equations are then derived from mass balance, coupled with empirical kinetics. Biological models are nonlinear and represent processes, which by essence are dynamic and adaptive. The temptation to embed most of the biology is high, with the risk that calibration would not be significant anymore. The most important task for a modeler is thus to ensure a balance between model complexity and ease of use. Since a model should be tailored to the objectives, which will depend on applications and environment, a universal model representing any possible situation is probably not the best option.

Here are 12 tips to develop your own bioprocess model. For more details on bioprocess modeling, the readers could refer to [1]. More tips concerning computational aspects can be found in [2, 3].

## Tip 1: Define your objective and the application context

**Fig 1:**
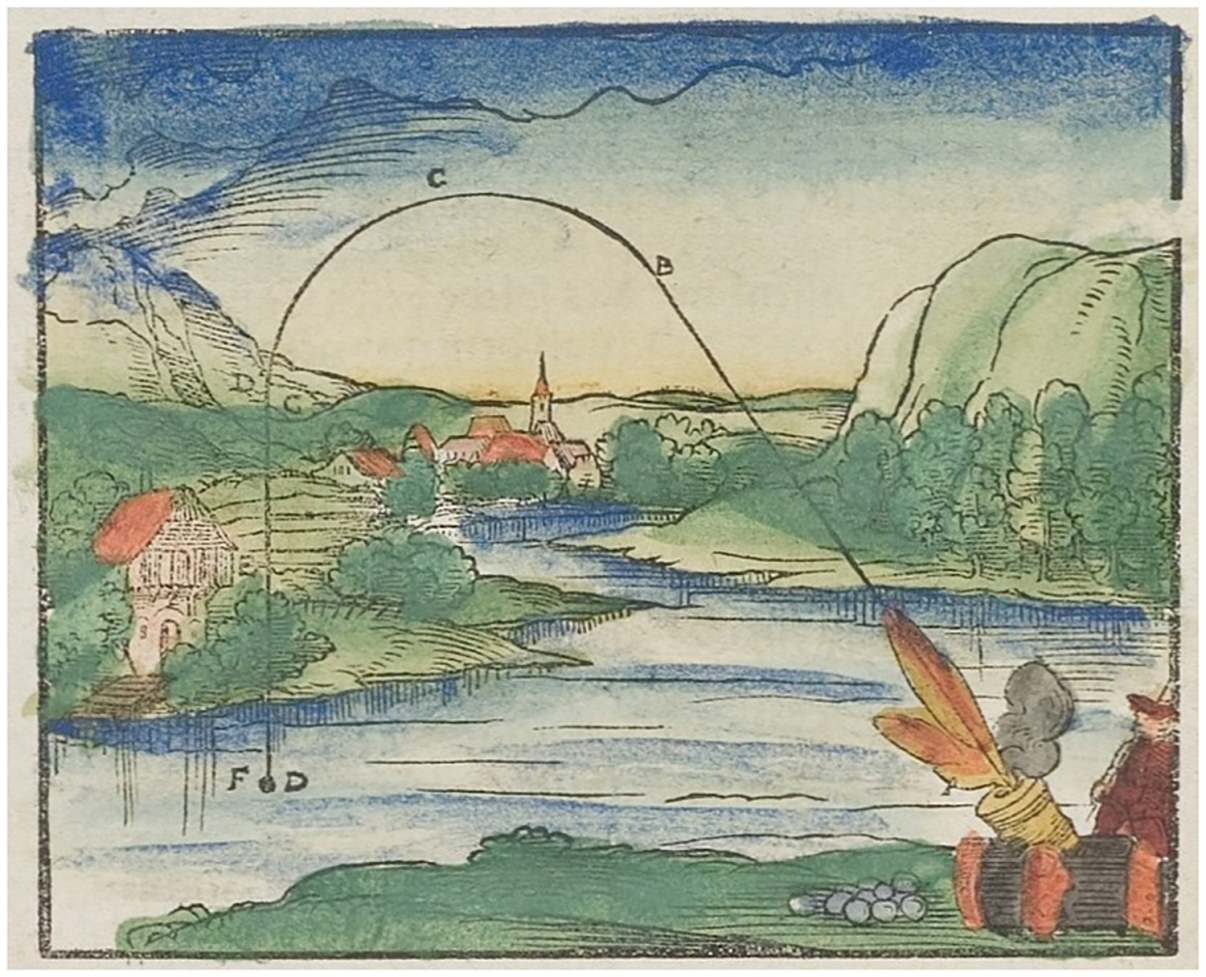
Medieval theory of the canon ball trajectory from Walther Hermann Ryff (1547) [6]. The canon ball trajectory was an assemblage of circular arcs and segments. Models in physics are now excellent approximations, but they have sometimes been improved during century-long periods. In biology, we are still at the dawn of model development.

Years of high school learning about how to set up mechanistic models based on the fundamental *F* = *m*.*a* relationship of mechanics or on the Ohm law have corrupted our minds. It took centuries to identify the corpus of laws supporting today’s physical models. Fig 1 recalls that, previously, there used to be some "less accurate" predictive models that have been forgotten. At present, models in these fields, even if empirical, are excellent approximations and—at least for those we studied at school—always ended up in rather simple, often linear, and mathematically tractable models. The complexity of biological systems requires a more open viewpoint, for which different models of the same process can be useful and complementary. Therefore, before writing equations, one must first clearly define the model objective. The model can be designed for numerous reasons, among them prediction of future evolution, understanding of the process behavior, estimation of unmeasured variables or fluxes, operator training, detection and diagnosis of failures, optimization, and control.

## Tip 2: Adapt your modeling framework with your objective, your knowledge, and your data set

When developing a model, it is crucial to keep in mind the objectives of the model and the framework for its application. A model targeting the understanding of some metabolic processes inherently requires the user to embark on the details of the cell metabolism [4, 5]. Predicting the impact of meteorology on outdoor microalgal processes means that light and temperature must be included somewhere in the model. A model for online control can be more straightforward (often because it will benefit from online information on process state). So keeping in mind the model objective, one has to choose which variables to include but also the type of model: deterministic versus stochastic, homogeneous versus heterogeneous (in terms of space or phenotype). The available data set or data that can be provided by the experimental setup will also constrain the choice of model complexity. Parameters should be calibrated at some point or at least reasonably determined from the experimental information. Model complexity can first be measured by the number of state variables (variables with dynamics) together with the number of parameters and stay compatible with the objectives and data.

## Tip 3: Take care with dimensions, intensive, and extensive properties

This tip seems very basic, but, in our opinion, it is worth emphasizing. The dimension of the model equation should be checked. Particular care should be taken between intensive and extensive variables [7]. This is particularly true when dealing with a metabolic model. A metabolite concentration could be expressed per unit of culture volume or intracellular volume. The concentration dynamics should then include the dilution by the reactor feeding or by cellular growth, respectively. Moreover, the kinetics of intracellular reactions should depend on intracellular concentrations, not culture concentrations. In several studies, it remains unclear.

## Tip 4: Do not assume gas concentrations equilibrate with atmosphere

Assuming gas concentrations equilibrate with the atmosphere is a common mistake. If we measure the dissolved CO_2_ concentration in a glass of water in equilibrium with the atmosphere, it will be proportional to $P_{CO_{2}}$, the CO_2_ partial pressure at the interface (i.e., in the air): $[CO_{2}]=K_{h}P_{CO_{2}}$ in which *K*_*h*_ is Henry’s constant at the considered temperature and salinity. At steady state, there is no more gas exchange between the atmosphere and liquid phase.

If algae are developing in the glass, the *CO*_2_ concentration will be lower, because the algae permanently consume it. As a consequence, there is a permanent flux of CO_2_ from air to water, with a flow rate

$$Q_{CO_{2}}=K_{L}a({\text{K}}_{\text{H}}{\text{PCO}}_{2}-CO_{2}),$$

which will balance the consumption of *CO*_2_ by the algae. Now the concentration of *CO*_2_ is lower than $K_{h}P_{CO_{2}}$, its natural equilibrium value without algae.

## Tip 5: Check the mathematical soundness of your model

A mathematical analysis of your model may help to detect potential errors, limitations, and drawbacks in model design and to better apprehend the process. Whenever possible, one should check mass conservations, check the boundedness of the variables (in particular their positivity), and study the asymptotic behavior of the model. This last point could be, for some models, particularly challenging. It is essential to keep in mind that nonlinear dynamical models are complex mathematical objects with potentially weird behaviors, including limit cycles, chaos, or abrupt change in behaviors after bifurcation when one of the model parameters has been slightly modified [8]. Mathematicians spend months trying to understand and prove the behavior of systems of low dimension, e.g., with "only" three state variables. The mathematical complexity is breathtaking when considering standard bioprocess models. Often, the properties of these models are hardly suspected, and Pandora’s box stays closed. Even the number of equilibria that can be produced is rarely discussed. Adding new features or including more realism into a model extends the risk of unexpected model behaviors.

The objective is to determine whether the trajectories of your system converge toward an equilibrium (a global equilibrium or different equilibria, depending on the initial conditions), if they present sustained oscillations (limit cycle) or even show a chaotic behaviour. These properties should be in line with the behavior of your bioprocess, otherwise the model should be revised.

## Tip 6: Be aware of structural identifiability

Most of the parameters in physical modeling have a clear meaning and can be directly measured on the process. Also, physical models are often linear. The theory of linear systems and their identification has received much attention; indirect identification of a tenth of parameters can be accurately carried out by modern algorithms [9, 10]. For the biological systems, which are in turn nonlinear and described by rough approximations, more modesty is required.

Theoretical identifiability of the parameters is a complex mathematical property [11], which is often characterized by cryptic (but accurate) mathematical formulations. In a nutshell, this theoretical mathematical property states that a parameter value can be uniquely determined by (nonlinear) combinations of measurements and their derivatives (with respect to time) at any order. More simply, a unique set of parameters can produce a given model output. With nonlinear models, it is possible that two sets of parameters can produce exactly the same results. To illustrate the nonidentifiability pathology, we present in Table 1 two illustrative astonishing examples for trivial models.

**Table 1:** Analysis of two simple examples with identifiability issues.

	Parameter set 1	Parameter set 2	False claim parameter meaning	Function
**Example 1**:	Substrate upake with inhibition		$\phi (S)=\bar{\mu }\frac{S}{S+K_{s}}\frac{K_{i}}{S+K_{i}}$	
Numerical values	$\bar{\mu }=2$ *K*_*i*_ = 1 *K*_*s*_ = 2	$\bar{\mu }=1$ *K*_*i*_ = 2 *K*_*s*_ = 1	Max. growth rate Inhibition constant Affinity constant	$\phi (S)=2\frac{S}{\left(S+1)(S+2\right)}$
General case	$\bar{\mu }$ *K*_*i*_ *K*_*s*_	$\bar{\mu }\frac{K_{i}}{K_{s}}$ *K*_*s*_ *K*_*i*_	Max. growth rate Inhibition constant Affinity constant	$\phi (S)=\bar{\mu }\frac{S}{S+K_{s}}\frac{K_{i}}{S+K_{i}}$
**Example 2**:	Logistic growth with mortality		$\dot{x}=\bar{\mu }(1-\frac{x}{K})x-Rx$	
Numerical values	$\bar{\mu }=2$ *K* = 1 *R* = 1	$\bar{\mu }=3$ *K* = 1.5 *R* = 2	Max. growth rate Carrying capacity Mortality rate	$\dot{x}=(1-2x)x$
General case	$\bar{\mu }$ *K* *R*	$\bar{\mu }+\theta$ $K\frac{\bar{\mu }+\theta }{\bar{\mu }}$ *R* + *θ*	Max. growth rate Carrying capacity Mortality rate	$\dot{x}=\bar{\mu }(1-\frac{x}{K})x-Rx$

In Example 1, two different parameter sets produce the same value of the function *ϕ*(*S*). In Example 2, an infinite number of parameter sets can produce the same dynamics $\dot{x}$ for an arbitrary value of *θ*. The parameters meaning (as often claimed) does then not make any sense.

The first example is unfortunately not so rare. It consists of representing an inhibition kinetics (from substrate *S*) with a product of Monod and a hyperbolic inhibition term. A numerical example is given in Table 1 (Example 1), in which two parameter sets produce exactly the same values. Parameters here are only locally structurally identifiable.

The second example in Table 1 uses a trivial logistic equation (*x* is the biomass) modified to deal with mortality rate (which is obviously a very bad idea). Here, an infinity of parameters provide the same biomass dynamics; they are structurally not identifiable.

These two examples also demonstrate that it is useless to attribute a biological meaning to a nonidentifiable parameter. In the first case, what was, in turn, the inhibition constant: *K*_*i*_ or *K*_*s*_? In the second example, is *K* the carrying capacity of the medium?

Perhaps more problematic when using an automatic algorithm for parameter identification, nonidentifiable parameters will kill any approach. Especially if it is a global approach, any optimization algorithm will oscillate between several of the possible solutions, or average them, and often will never converge.

In general, assessing identifiability for complex dynamical models is very challenging. This is a reason why modelers must refrain from embedding too many processes into a model and privilege lower complexity models when only a limited set of measurements is available for validation.

## Tip 7: Double check numerical implementation

If your model has been implemented only once, then it probably contains at least three mistakes. We know this is not true for you, but it is for most of the people. So if the model was right, after a rapid change in one of the equations for testing the effect of one factor, it would become wrong because eventually the test is not removed. There are strict coding rules and use of validation tests [12], but they are rarely respected for model development because the model implementation is generally not carried out by computer scientists. Also, the way models are implemented can highly differ, and some computer languages may be more difficult to cross check. Excel is an excellent tool for displaying data and for simple computations, but it is not an appropriate tool for simulating complex models since it is almost impossible to cross-check implementation. Some graphical languages also have these drawbacks when a connection to a wrong node can corrupt the result while being almost impossible to detect.

One way of reducing the risk of error is a double implementation, with two different computer programmers and two different languages. This has been the case for the models used in wastewater treatment, Anaerobic Digestion Model No.1 (ADM1) for anaerobic digestion [13], and Activated Sludge Model No. 1 (ASM1) for activated sludge [14]. The first comparison between different implementations revealed to be quite quaint. Also, simple case studies must help to check simple theoretical properties (positivity of variables, mass conservation, etc.) that must be respected.

## Tip 8: Pay attention to practical identifiability

The cost criterion to be optimized (typically the sum of squared errors) is generally nonconvex, and many local minima perturb parameter identification. In practice, it is often not possible to get an accurate estimate of parameters from the data sets. The most efficient algorithms are generally limited to three parameters to be determined per measured quantity (assuming a reasonable sampling over time). The weird consequence is that fitting a model to a set of data is generally possible but that does not mean that the estimated parameters are reasonable. Whenever a parameter has a clear meaning, the validity of the identified value must always be checked, and bounds can be added during the identification process. Multiple algorithm initialisations are also strongly recommended. Collecting informative data is also key for practical identifiability, which means data corresponding to high sensitivities of the model outputs with respect to parameter variations (cf. Fisher information matrix [9]). As a matter of illustration, it is not possible to estimate a parameter related to growth inhibition if substrate concentration is always too low to trigger inhibition.

Finally, a literature review is an essential resource for parameter values, in particular for algorithm initialisation. Nonetheless, exotic chimaera can appear when picking up parameters from different papers.

## Tip 9: Apply the "divide and conquer" strategy to identify your parameters

Do not try to get all your parameters at once, through a never converging optimization algorithm and rather identify subsets of parameters. In many cases, after simple algebraic manipulations, some parts of the model can lead to relationships between some measured quantities and eventually provide some combinations of the parameters. For example, the pseudo-stoichiometry can often be identified independently of the reaction rates after some straightforward transformations [15]. Some working modes do considerably simplify the model and are often an opportunity to extract such relationships. For example, during a phase when nutrients are nonlimiting, the Michaelis–Menten kinetics can be replaced by constants. Similarly, if different equilibria can be observed for various inputs, they would probably lead to very interesting relationships between some of the model parameters [16].

## Tip 10: Determine parameter and model uncertainties

Assessing measurement uncertainty propagation is of utmost importance to assess model accuracy. This first means that the experimental data must be associated to the variance of their measurement error. There are different strategies to compute not only the parameter values but also their confidence intervals. This is straightforward when parameters are deduced from linear relationship but can also be estimated in a more complex case thanks to the covariance matrix of parametric errors [9]. The strong scientific added value is that the simulation scheme will predict not only outputs but also the confidence intervals derived from the covariance matrix of prediction errors or from Monte Carlo simulations.

## Tip 11: Validate the model with data not used for identification

When observing the vast diversity in bioprocess models, only a few of them have been appropriately validated. First, because it is not possible to validate a model, a model can only be discarded when it is not compliant with experimental records [17]. However, assuming a relaxed use of the "validation" term, it would mean that the model has been proven accurate for a large variety of cases, particularly for cases significantly different from the learning data set (data that has been used for the calibration). This ideal situation is very difficult to meet in practice, and most of the time, the validation data sets only differ by some initial conditions or by a single different forcing variable. If the model has enough parameters, it can probably fit a calibration data set nicely with only a few points. However, it will exhibit abysmal performances for cross-validation. For larger calibration data sets, the fit will probably less successfully highlight the quality of the model, but prediction capacity might be highly enhanced. The plot will not look that nice, but the model will definitely be more powerful and relevant.

Claiming that the model is valid is, therefore, an act of faith, and a very weak scientific assertion. As running experiments takes time and is money consuming, the number of experiments is, by essence, limited. As a consequence, it becomes clear that the conditions for which the model has been validated must be clearly stated. Knowing the "model validation domain" will in itself be precious for future model use. Also, providing data sets for which the model did not do its job is intrinsically useful, although rarely done.

Often, the question is instead to choose the best model among a few candidates. A more complex model, with more parameters, will mechanically better fit the data. However, that does not mean it is more correct, it just means it is more flexible. The Akaike criterion [18] is a good option to compare the performance of two models of different levels of complexity. However, the only real criterion to assess the predictive power of a model, and therefore to compare model performances is cross-validation, assessing the model with data that were not used for calibration (and data whose dynamics are significantly different from the calibration data set). Additionally, the candidate models can even be used to find the experimental conditions that will allow to differentiate them better [17].

Finally, models can include the effects of different factors, which often have been studied separately. The models then gather these effects classically by multiplying the different terms or using Liebig’s law of minimum. Validation experiments could be the last chance to test possible interactions between these factors and find the best way to combine their effects in the model.

## Tip 12: Share codes, tips, tools, and model limitations

More and more journals require this, and it is to be welcomed. Providing your model—with all the files necessary to reproduce your simulations (including parameter values, initial conditions, etc.)—will favor its dissemination within the scientific community. Your model would thus be further validated with new data sets. Additionally, it promotes error checking, helps the reader if some model details in the manuscript are unclear, and removes any suspicion of fraud.

More generally, what makes the success and the efficiency of a model is not limited to the biology it embeds and to the realism of its predictions. A model is inexorably associated with a set of tools to calibrate it, estimate which are the most sensitive parameters, optimize a criterion, determine the input which maximizes productivity, etc. The associated toolbox to make the model applicable and efficient is probably at least as necessary as the model itself. Great models can have complex structures or behaviors, which eventually make their use more tricky. For example, the outstanding Geider model [19] is in turn rather challenging to calibrate, and specific methods dedicated to its calibration are needed [20]. Even simpler models, such as the Hinshelwood model [21] for temperature, advantageously predicts a mortality rate [22], but calibrating this model often turns into a nightmare [23]. Keeping two different modeling approaches can significantly help in this case by using the toolbox of one of the models to manage the other one. Typically, using a temperature response model from [24] as a gauging device makes the calibration of Hinshelwood’s model much less painful. Providing all these kinds of information on your model should promote its adoption by the community.

## Conclusion

Modeling in biology is a question of choices and trade-offs. The striking difference between two different modelers is often the choice in model complexity. Extensive tests, using cross-validation data sets or based on Akaike criteria, may reveal that one model has a better prediction capability than the other, but in other circumstances, it might be the opposite. Our culture has contributed to hatch the illusion of a unique and universal model behind nature. However, even if this idea was right, we are far from having discovered it. Also, always trying to run after such universal representations of nature inexorably leads to models whose complexities do not match the available measurements and our capability to validate the model. So, why should we keep a unique model? Why not use a series of models of increasing complexity? Surrogate models consist of a simplified version of a simulator, which is easier to handle mathematically, resulting in more straightforward use for optimization or control. The surrogate model can be derived and calibrated from the most complex model, but the opposite is also true. A simplified model, with limited accuracy, can provide bounds for a more detailed model. Also, a complicated model can be simplified into different submodels depending on the environment and the limiting factor (nutrients, light, or temperature). Working with a set of coherent models should not necessarily increase difficulty, it creates a consistent framework that can prove to be very useful for different purposes, from model calibration and process optimization to advanced control.
